# Clinical and Molecular Characteristics of 100 Atypical Teratoid Rhabdoid Tumor Patients from Low- and Middle-Income Countries

**DOI:** 10.3390/cancers17183077

**Published:** 2025-09-20

**Authors:** Noha A. Ismail, Shaimaa Aboubakr, Amal Mosaab, Eslam Maher, Hanafy Hafez, Hala Taha, Dina Yassin, Amal Refaat, Mohamed S. Zaghloul, Mohamed El-Beltagy, Abdelrahman Enayat, Volker Hovestadt, Olfat Ahmed, Mark W. Kieran, Ahmed El-Hemaly, Shahenda El-Naggar, Alaa El-Haddad

**Affiliations:** 1 Pediatric Oncology Department, Children’s Cancer Hospital Egypt 57357, Cairo 11441, Egypt; noha.ismail@57357.org (N.A.I.); shimaa.abobakr@57357.org (S.A.); hhafez75@cu.edu.eg (H.H.); mark.kieran@dayonebio.com (M.W.K.); alaa.hadad@57357.org (A.E.-H.); 2 Tumor Biology Research Program, Basic Research Unit, Research Department, Children’s Cancer Hospital 57357, Cairo 11441, Egypt; amal.abdelaziz@57357.org; 3 Department of Clinical Research, Children’s Cancer Hospital Egypt 57357, Cairo 11441, Egypt; e.maher@imperial.ac.uk; 4 Department of Surgery and Cancer, Faculty of Medicine, Imperial College London, London SW7 2AZ, UK; 5 Department of Pediatric Oncology, National Cancer Institute, Cairo University, Cairo 12613, Egypt; 6 Department of Pathology, Children’s Cancer Hospital Egypt 57357, Cairo 11441, Egypt; hala.taha@57357.org; 7 Department of Pathology, National Cancer Institute, Cairo University, Cairo 12613, Egypt; 8 Department of Clinical Pathology, Molecular Biology, Children’s Cancer Hospital Egypt 57357, Cairo 11441, Egypt; dina.yassin@57357.org; 9 Department of Radio Diagnosis, Children’s Cancer Hospital Egypt 57357, Cairo 11441, Egypt; amal.mohamed@57357.org; 10 Department of Radio Diagnosis, National Cancer Institute, Cairo 12613, Egypt; 11 Department of Radiation Oncology, Children’s Cancer Hospital Egypt 57357, Cairo 11441, Egypt; mohamed.zaghloul@57357.org; 12 Department of Radiation Oncology, Kasr Al-Ainy School of Medicine, Cairo University, Cairo 12613, Egypt; 13 Department of Neurosurgery, Children’s Cancer Hospital Egypt 57357, Cairo 11441, Egypt; mohamed.elbeltagy@kasralainy.edu.eg (M.E.-B.); abdelrahman.enayat@57357.org (A.E.); 14 Department of Neurosurgery, Kasr Al-Ainy School of Medicine, Cairo University, Cairo 12613, Egypt; 15 Department of Pediatric Oncology, Dana-Farber Cancer Institute, Boston, MA 02115, USA; volker_hovestadt@dfci.harvard.edu; 16 Division of Hematology/Oncology, Boston Children’s Hospital, Boston 02115, MA, USA; 17 King Hussein Cancer Center (KHCC), Amman 11941, Jordan; olfat.ahmed@kitz-heidelberg.de; 18 Division of Pediatric Neuro-Oncology, Hopp Children’s Cancer Center (KiTZ), 69120 Heidelberg, Germany

**Keywords:** ATRT, molecular, prognostic, low-middle income, outcome

## Abstract

Background: Atypical teratoid rhabdoid tumor (ATRT) is a highly aggressive, rare pediatric central nervous system malignancy. Prognostic factors for optimizing risk stratification and management in a large uniformly treated cohort are lacking. Methods: We conducted a single-center retrospective cohort study analyzing clinical and outcome data for 100 newly diagnosed ATRT patients aged <18 years treated at the Children’s Cancer Hospital, Egypt, from 2008 to 2022. They were treated uniformly as per the Dana-Farber Cancer Institute modified IRS-III protocol. Molecular subgroups (MYC, SHH, and TYR) were determined via a DNA methylation array for patients who had sufficient DNA material available for the methylation analysis. Treatment toxicities were graded per the Common Terminology Criteria for Adverse Events (CTCAE) v5.0. Results: The median age at diagnosis was 1.88 years (IQR 0.99, 3.01); 28% were under 1 year of age, 45% were between 1 and 3 years old, and 26% were above 3 years of age. At diagnosis, 39% of patients had metastatic disease. A total of 60% of patients had gross residual disease following surgical excision. In multivariable analysis, age < 1 year and metastatic disease had a significant impact on event-free survival (EFS) (*p* = 0.047 and *p* = 0.002, respectively); however, only metastatic disease had a significantly negative effect on overall survival (OS) and cumulative incidence of relapse (CIR) (*p* = 0.002 for OS and *p* < 0.001 for CIR). DNA methylation was performed for 69 patients who were classified as having a TYR (n = 13), SHH (n = 34), MYC (n = 17), or non-ATRT diagnosis (n = 5). In the cohort of the 64 patients with ATRT defined by methylation, no significant survival differences were observed. Treatment-related deaths were reported in 28% of our studied group. Gram-negative septicemia was the most common cause of toxic death. The 5-year EFS and OS of the whole cohort were 12% and 13%, respectively. Conclusions: In this cohort, no significant survival differences were observed among the methylation subgroups. The higher treatment-related mortality in our cohort compared to the original protocol’s toxic-related deaths suggested that intensive and lengthy chemotherapy regimens may need modification for our population. The need for a short intensified approach, including a limited induction cycle followed by an intensified high-dose consolidation therapy, may be more appropriate for our patients with low socioeconomic status to avoid a repeated and prolonged course of protracted neutropenia.

## Simple Summary

This retrospective cohort study analyzed 100 pediatric patients with atypical teratoid rhabdoid tumors (ATRTs) treated at Children’s Cancer Hospital, Egypt, between 2008 and 2022. Patients were managed with the Dana-Farber Cancer Institute modified IRS-III protocol, and molecular subgrouping was performed retrospectively. Event-free survival and overall survival at 5 years were 12% and 13%, with infants (<1 year) and patients with metastatic disease experiencing the poorest outcomes. The extent of resection correlated with survival, while molecular subgroups (MYC, SHH, TYR) showed no significant prognostic impact. Treatment-related mortality was notably high (28%), predominantly from Gram-negative septicemia. These findings suggest that while ATRT remains a highly aggressive malignancy with poor outcomes in low- and middle-income countries, modifying intensive and prolonged chemotherapy regimens toward shorter, more intensified approaches may improve both survival and treatment tolerability in this vulnerable population.

## 1. Background

Atypical teratoid/rhabdoid tumors (ATRTs) are aggressive malignancies of the central nervous system (CNS), predominantly affecting children aged three years or younger [1,2]. The mortality rate of this age group is approaching 70% [3]. These tumors are typically large and invasive and frequently present with metastatic disease at diagnosis, reported in up to 40% of cases [4].

The hallmark genetic alteration in ATRT is the bi-allelic inactivation of the *SMARCB1* gene and, less commonly, *SMARCA4* [3,5]. These tumor suppressor genes encode essential components of the SWI/SNF chromatin remodeling complex, which regulates cell proliferation and differentiation [4,6]. Notably, up to one-third of ATRT patients harbor germline *SMARCB1* or *SMARCA4* mutations, predisposing them to multiple intra- and/or extracranial rhabdoid tumors at an early age [7].

Molecular heterogeneity has been well-characterized in ATRT. Based on DNA methylation profiling, three distinct molecular subgroups have been identified: ATRT-SHH, ATRT-TYR, and ATRT-MYC [8]. Each subgroup exhibits unique epigenomic and transcriptomic signatures, as well as different clinical characteristics, including differences in age at diagnosis, tumor location, and neuroimaging features. However, the prognostic and therapeutic implications of these molecular subgroups remain under investigation [8].

Despite advances in aggressive multimodal therapy, including surgery, chemotherapy, and radiotherapy, survival outcomes for ATRT remain variable, with reported 5-year overall survival (OS) rates ranging from 15% to 70% across different cohorts [1,9].

Several prognostic factors have been proposed, including young age at diagnosis, metastatic disease, incomplete surgical resection, high-dose chemo/radiotherapy, and germline *SMARCB1*/*SMARCA4* alterations. However, there remains no consensus on which factors most significantly influence prognosis or should guide risk-adapted therapy [1,10].

The Dana-Farber modified IRS-III protocol, which incorporates surgery, systemic and intrathecal chemotherapy, and radiotherapy, has shown promising survival improvements in patients with ATRT [10]. Nevertheless, treatment-related toxicity and mortality remain underreported, especially in real-world settings in low- and middle-income countries (LMICs).

A prior study by El-Hemaly et al. [11], which included 47 ATRT patients treated at the Children’s Cancer Hospital, Egypt (Cairo), between July 2007 and December 2017, revealed a treatment-related mortality rate of 29.8%, significantly higher than that reported by Chi et al., who documented only one toxic death among 20 patients [10].

This study aimed to evaluate the prognostic impact of various clinical and molecular factors, including age, tumor location, metastatic status, extent of resection, early treatment response, germline mutation status, and molecular subgroup, on ATRT outcomes.

## 2. Patients and Methods

A single-center retrospective cohort study was conducted analyzing 100 ATRT patients diagnosed by histopathology (morphology and immunohistochemistry loss of SMARCB1 gene product integrase interactor 1 (INI1) protein) and aged <18 years treated at the Children’s Cancer Hospital, Egypt, 57357, from 2007 to 2022. Maximal safe resection was attempted at presentation, and second-look surgery was supposed to be performed after induction chemotherapy for patients with residual disease. According to the neurosurgeon’s assessment, none of those patients underwent second-look surgery, as it is a risky procedure.

The extent of surgical resection was defined as gross total resection (GTR), near-total resection (NTR) (>90% of tumor excised), subtotal resection (STR) (<90% of tumor excised), or biopsy based on postoperative magnetic resonance imaging (MRI) and the surgeon’s intraoperative assessment [12]. Metastatic staging was performed with an MRI of the brain and spine and lumbar puncture for cerebrospinal fluid (CSF) analysis.

The Dana-Farber Cancer Institute modified IRS-III chemotherapy protocol was implemented for the whole cohort. The induction phase consisted of 6 weeks of chemotherapy. The consolidation phase consisted of chemo-radiotherapy from week 7 to 18 and the maintenance phase from week 19 to the end of the protocol, with an interval of 3 weeks between each cycle of chemotherapy. See Figure S1.

Response to chemotherapy was assessed after 6 weeks of treatment. It was classified as complete response (CR), partial response (PR), stable disease (SD), or progressive disease (PD) [10].

Radiotherapy was given at week 7 of induction chemotherapy as conformal radiation for patients with non-metastatic disease (M0). Those with metastatic disease (M+) and aged <3 years old received a tumor-directed dose of 54 Gy. Patients with M+ disease who were older than 3 years of age received craniospinal irradiation (CSI) at a dose of 36 Gy with a 54 Gy boost to the tumor bed.

Grade 3 and higher therapy-related toxicities were collected according to the common terminology criteria for adverse events (CTCAE) Version 5.0 [13]. We reported hematological toxicities (including anemia and febrile neutropenia), nephrotoxicity, neurotoxicity, gastrointestinal toxicity, and metabolic disturbances (hyponatremia, hypokalemia, and hypomagnesemia) throughout different treatment phases.

### 2.1. Multiplex Ligation-Dependent Probe Amplification (MLPA)

Twenty-eight blood samples were obtained for the germline testing of the SMARCB1 gene either from the blood biorepository bank or directly from patients after consenting. DNA was isolated using the Gentra Pure Gene kit ( Sigma-Aldrich, St. Louis, MO, USA) and tested for the deletion or duplication of the SMARCB1 gene using SALSA MLPA probemix P25B SMARCB1 (Marc Holland, Amestrdam, Netherland).

### 2.2. Genome-Wide DNA Methylation Profiling

Genomic DNA extraction from the FFPE samples was performed as previously described [14]. In brief, DNA isolation was performed using the QIAamp DNA FFPE Tissue Kit ( Sigma-Aldrich, St. Louis, MO, USA). DNA quantification was performed using the DENOVIX Fluorometer (ds DNA High Sensitivity) (Merck, Darmastadt, Germany. DNA quality was assessed using the Illumina FFPE QC kit (Illumina Inc., San Deiago, CA, USA). An EZ DNA methylation kit (D5002, Zymo Research, Orange, CA, USA) was used for the bisulfite conversion of the extracted DNA using a minimum of 250 ng. Bisulfite-converted DNA was restored using the Infinium HD FFPE DNA Restore Kit (WG-321-1002, Illumina Inc., San Deiago, CA, USA), followed by hybridization to Illumina Infinium Human Methylation EPIC 850K bead chips. Hybridized chips were then scanned using the Illumina iScan microarray scanner according to the manufacturer’s recommendations (Illumina Inc., San Deiago, CA, USA).

### 2.3. Methylation Analysis

Methylation analysis was performed using the minfi Bioconductor package v. 1.29.3. Raw signal intensities were obtained from Illumina intensity data (IDAT) files. Samples were normalized using Noob. Beta values were obtained using single-sample Noob normalization (preprocessNoob). Probe filtration included probes located on sex chromosomes and probes containing SNPs (n = 24,747) in their bodies or at a CpG, with a minor allele frequency (maf) > 0.05. Unsupervised hierarchical clustering was performed using the cor function and hclust for clustering analysis, with the average distance as the clustering metric.

Methylation-based tumor classification was performed using the Heidelberg classifier (v12.8), based on the recently published random forest classification algorithm [15]. Tumor samples were projected onto a suitable reference cohort from the Molecular Neuro-Pathology (MNP) reference set from the German Cancer Research Center (DKFZ). A t-distributed stochastic neighbor embedding (t-SNE) plot was initially generated using the DKFZ’s in-house Shiny application and further edited using RStudio (version 4.2.0) and Inkscape 1.3.2 [16].

### 2.4. Statistical Methods

Overall survival (OS) was defined as the time from presentation to death from any cause or the last contact. In contrast, event-free survival (EFS) was defined from presentation to the date of relapse, progression, or death from any cause (i.e., any event). Survival probabilities were computed with the Kaplan–Meier estimator alongside a 95% confidence interval (CI) using the log method. OS and EFS were modeled using Cox regression. Acknowledging that a considerable number of deaths were not disease-related (toxic deaths), we accounted for this competing risk to delineate the hazards of relapse and related mortality from non-relapse mortality (NRM). For this, an analysis of the cumulative incidence function (CIF) and fitted sub-distribution hazards with a Fine–Gray regression for the event of interest (Relapse) was used. No missing data imputation was performed, and missing baseline M-stage values were included as parameters in our models (Mx). A sensitivity analysis was conducted through complete case analysis in multivariable models by omitting Mx cases. Another sensitivity analysis regarding the handling of competing risks used the cause-specific Cox model approach by censoring NRM, defined as progression-free survival (PFS). Neither sensitivity analysis changed the results. R version 4.4.2 was used, with packages ‘survival’ v3.7-0, ‘cmprsk’ v.2.2-12, and ‘tidycmprsk’ v1.1.0.

## 3. Results

### 3.1. Patient Characteristics

This study involved 100 ATRT patients with a median age of 1.88 years (IQR 0.99–3.01). Among them, 28 patients were under 1 year of age, 46 patients were between 1 and 3 years, and 26 patients were older than 3 years, with a male-to-female ratio of 1.4:1. A total of 41 patients had supra-tentorial tumors, 55 had infra-tentorial primary tumors, and 4 patients had spinal ATRT. Thirty-nine patients presented with metastatic disease. A total of 40 patients underwent GTR/NTR, 29 patients had STR, and 31 patients underwent biopsy. None of the 24 patients with residual disease at week 6 of chemotherapy underwent second-look surgery. See Table 1.

For the germline mutation of the SMARCB1 gene, blood samples were available for 28 patients; 9 samples were excluded due to poor DNA quality. A total of three patients were positive for mutation (one with a deletion at exon 4, one with a duplication at exon 9, and one with a duplication at exon 1). In comparison, 16 patients did not have any germline mutation.

**Figure 1:**
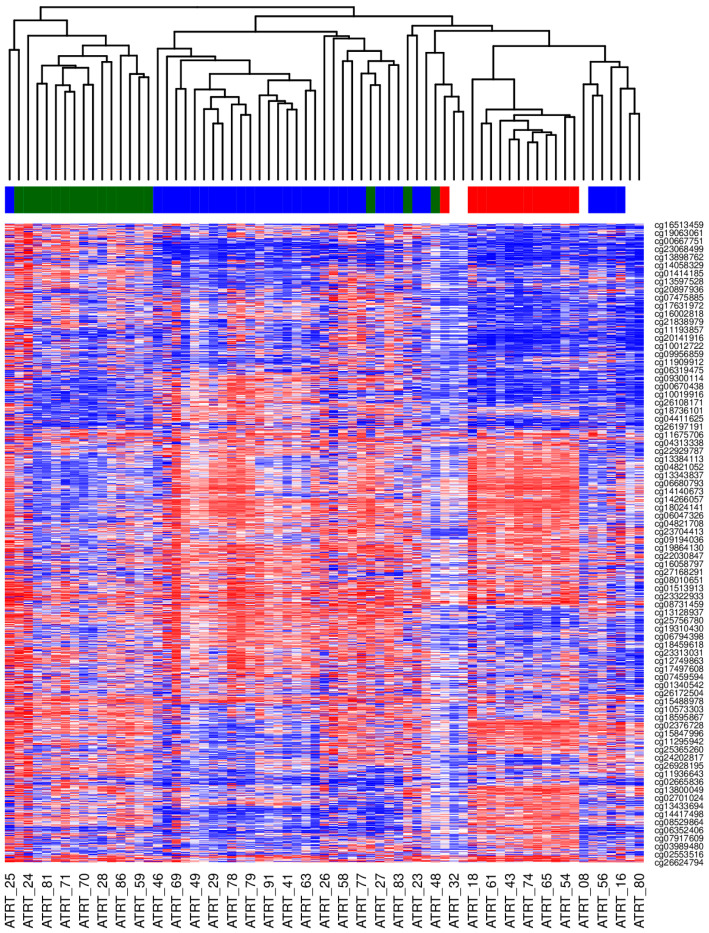
A heatmap showing the unsupervised hierarchical clustering of ATRT samples from CCHE (Cairo) using beta values for the most variable CpGs. ATRT-TYR = red; ATRT-SHH = blue; ATRT-MYC = dark green. Only every second sample is annotated on the *X*-axis.

**Figure 2:**
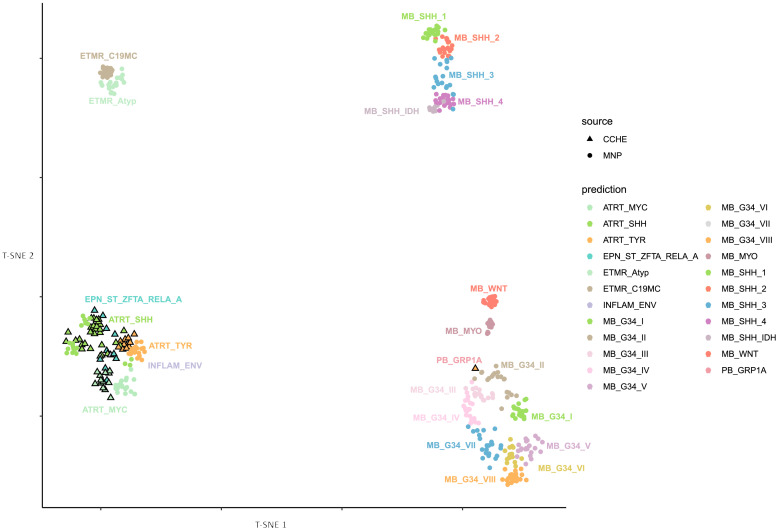
t-SNE plot illustrating DNA methylation-based classification (v12.8) of ATRT cohort from CCHE, Cairo, in comparison with MNP reference set from Heidelberg.

Tumor DNA methylation profiling was performed for 69 patients with a histologically confirmed diagnosis of ATRT (based on SMARCB1 loss by immunohistochemistry). Using the MNP classifier (version 12.5), 64 cases were classified as ATRT, distributed into the known molecular subtypes: 34 as ATRT-SHH, 17 as ATRT-MYC, and 13 as ATRT-TYR. See Table S2. Unsupervised clustering analysis based on the most variable CpGs confirmed these assignments, grouping the samples into three distinct clusters that aligned perfectly with the TYR, SHH, and MYC subtypes, as shown in Figure 1. The v12.5 classifier assigned the remaining five cases to other entities: two as supra-tentorial ependymoma, ZFTA fusion-positive; one as medulloblastoma, group 3; one as medulloblastoma, SHH; and one as chordoma with very low scores. To further confirm the status of these cases, we remapped the entire cohort using the latest MNP classifier (version 12.8). This reanalysis resulted in an additional seven cases as supra-tentorial ependymoma (ZFTA fusion-positive) and one as pineoblastoma (PB-GRP1A) despite originally being SHH with a score of 0.9, as seen in Figure 2. Given that all cases were selected based on the pathological feature of ATRT (SMARCB1 loss), we excluded the five cases from the subsequent analysis because they were classified as non-ATRT by both classifier versions.

### 3.2. Chemotherapy

**Figure 3:**
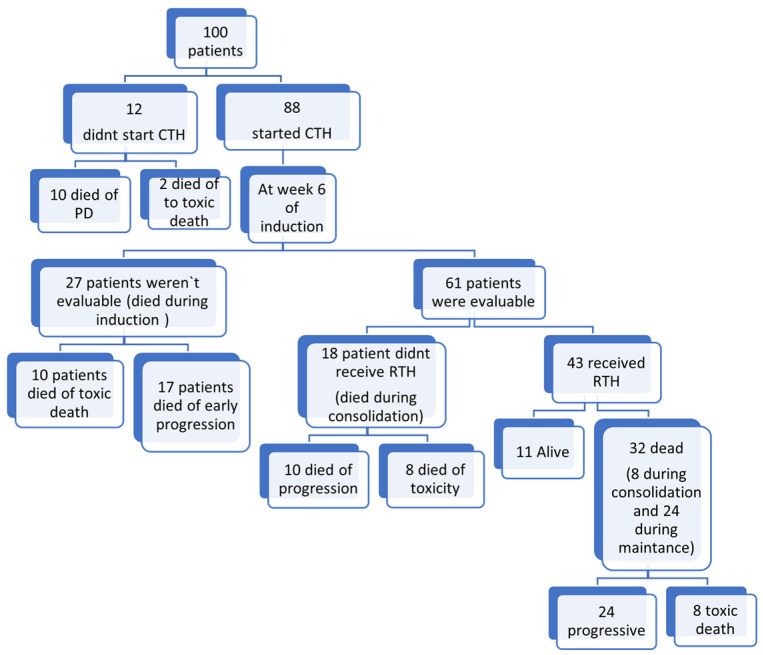
A flow chart of the whole patient cohort. Abbreviations: PD: progressive disease; CTH: chemotherapy; RTH: radiation therapy.

The median time to start chemotherapy post-surgical excision was 26 days (range 4–120 days). Twelve patients did not receive chemotherapy; one patient died secondary to a shunt infection with a Gram-negative multidrug-resistant organism, and eleven patients died secondary to advanced disease or progression within 30 days from surgery before chemotherapy commencement. Only 21 patients completed the full protocol treatment. In total, 20 patients died secondary to sepsis, while 37 patients died secondary to disease progression, as shown in Figure 3.

### 3.3. Response to Induction Treatment

A total of 61 patients were evaluable after 6 weeks of induction chemotherapy, 30 patients were in CR, 16 patients had PR, 8 patients showed SD, and 7 patients had PD.

### 3.4. Radiotherapy

The median time to radiotherapy initiation was 116 days (range 79–233 days) from the start of chemotherapy. Radiotherapy was delivered to 43 patients. A total of 7 patients received CSI with a boost to the tumor bed, and 36 patients received focal radiation therapy (32 M0, 4 M+). Fifty-seven patients were unable to receive RTH. A total of 20 patients died of sepsis, and 37 patients died of progressive disease before RTH was to be delivered. See Figure 3.

### 3.5. Toxicity and Treatment Complications

Grade 3 hematological toxicities were more evident during the induction phase; 76 patients had grade 3 anemia, and 57 patients developed grade 3 fever and neutropenia. Grade 5 fever and neutropenia were reported in 13 patients during induction, 1 patient during consolidation, and 2 patients during the maintenance phase.

Grade 3 and 4 metabolic toxicities (hypokalemia, hyponatremia, hypomagnesemia, and hypocalcemia) were common. They included grade 4 hypokalemia in 61 patients during the induction phase, 12 patients during the consolidation phase, and 8 patients during the maintenance phase.

Regarding the gastrointestinal side effects, 12 patients developed grade 3 oral mucositis, and 7 patients had grade 3 diarrhea. Grade 5 diarrhea was reported in one patient who died from shock during the induction phase. Grade 3 third nerve palsy due to vincristine toxicity was reported in four patients during the induction phase and in one patient during maintenance. No other cranial nerve palsies were reported. See Table S1.

### 3.6. Methylation Groups

Patients with a TYR signature were significantly younger than those with ATRT-MYC and ATRT-SHH, with median ages of 0.97 years (IQR 0.58–1.36), 2.44 years (IQR 1.53–3.00), and 1.91 years (IQR 1.05–2.96), respectively.

ATRT-TYR was exclusively presented in the infra-tentorial region, whereas ATRT-SHH was exhibited in both supra-tentorial and infra-tentorial locations in 44% and 56% of the cohort, respectively. MYC-ATRT had a supra-tentorial location (53%) and an infra-tentorial location (24%). All patients with spinal involvement had the ATRT-MYC epigenetic group.

Metastatic disease was documented in 6, 13, and 3 patients within the MYC, SHH, and TYR groups, respectively. Gross total resection was achieved in 5, 20, and 6 patients within the MYC-ATRT, SHH-ATRT, and TYR-ATRT groups, respectively.

Regarding the response at week 6 of induction chemotherapy, CR, PR, and PD were reported in six, five, and one patient within the MYC group, respectively. The SHH ATRT group had 15 patients with CR, 3 with PR, and 3 with PD. Finally, within the TYR group, four patients had complete remission, and two showed partial remission with no documented progressive disease. See Table S2.

### 3.7. Survival Analysis

Age had a significant impact on survival, as patients < 1 year of age had the worst 5-year EFS and OS of 0%; patients aged 1–3 years had a 5-year EFS and OS of 13% and 15%, respectively (*p* = 0.02 for EFS; *p* = 0.03 for OS); and patients aged >3 years had a 5-year EFS and OS of 23% for both (*p* = 0.01 for EFS; *p* = 0.02 for OS). The cumulative incidence of relapse (CIR) did not differ between the three age groups; it was 64% for patients aged <1 year, 59% for patients aged 1–3 years, and 62% for patients aged >3 years (HR 0.9, 95% CI 0.45–1.8, *p* = 0.7 for age > 3 years and HR 0.8 95% CI 0.4–1.4, *p* = 0.4 for age 1–3 years compared to age < 1 year).

The primary tumor site had a borderline impact on survival, with 5-year EFS and OS rates of 20% and 6.8% for supra-tentorial and infra-tentorial locations, respectively (*p* = 0.05 for EFS and *p* = 0.05 for OS). The 5-year CIR was 61% for both supra-tentorial and infra-tentorial locations (HR = 1, 95% CI 0.6–1.6, *p* = 0.9).

Patients with metastatic disease had a 5-year EFS and OS of 0% compared to the non-metastatic disease group with an EFS and OS of 23% and 25% (*p* < 0.001 for both EFS and OS). The relapse risk was significantly higher in patients with M+ compared to their M0 counterparts with a CIR of 87% and 43%, respectively (HR = 4, 95% CI 2.5–7, *p* = 0.001). See Figure S2.

Patients with residual disease had a worse 5-year EFS and OS of 6% for both compared to patients with GTR with a 5-year EFS and OS of 20% and 22%, respectively (*p* = 0.018 for EFS and *p* = 0.021 for OS). Patients with residual disease were at a higher risk of relapse at 5 years compared to those with GTR, with a CIR of 75% and 40%, respectively (HR = 2.8, 95% CI 1.6–4.9, *p* < 0.001). See Figure S2.

There was no significant survival difference between the three molecular groups. The 5-year EFS was 18%, 12%, and 7% for MYC, SHH, and TYR, respectively, with *p*-values of 0.9 and 0.3 for SHH and TYR, compared to the MYC group. The 5-year OS rates were 18% and 15% for MYC and SHH, respectively. Notably, no patients with TYR were alive at 5 years, with *p*-values of 0.6 and 0.4 for SHH and TYR, respectively, compared to the MYC group. Additionally, molecular subgrouping did not confer any prognostic value regarding the cumulative incidence of relapse, as the 5-year CIR was 53%, 68%, and 46% for MYC, SHH, and TYR, respectively. The *p*-values for SHH and TYR were 0.5 and 0.6, respectively, compared to the MYC subgroup.

In multivariable analysis, age and metastatic disease had a significant impact on EFS (*p* = 0.047 and *p* = 0.002, respectively); however, only metastatic disease had a significant negative impact on OS and CIR (*p* = 0.002 for OS and *p* < 0.001 for CIR)—Table 2, Table 3 and Table 4.

### 3.8. Pattern of Relapse/Progression

Out of 61 patients who showed progression or relapse, local failure was documented in 13, distant failure in 18, and combined distant and local failure in 30.

Of the 32 patients with M0 disease who received focal radiation therapy, 16 relapsed. Among these, 12 patients (75%) experienced distant relapse, 2 patients (12.5%) had local failure, and 2 patients (12.5%) had both local and distant relapse. Nine of the sixteen relapsed patients were ATRT-SHH-type: six had distant relapse, two had local relapse, and one had both local and distant relapse. Two patients were ATRT-TYR-type, and two were ATRT-MYC-type; all of them had distant relapse.

Four patients who had metastatic disease and were younger than 3 years received focal radiation therapy; all of them experienced relapse. Two patients had distant and local relapse, one patient had distant relapse, and one patient had local relapse. The median follow-up duration was 5.2 years (IQR: 4.5 to 8) for the entire cohort.

### 3.9. The Outcome of the Whole Cohort

**Figure 4:**
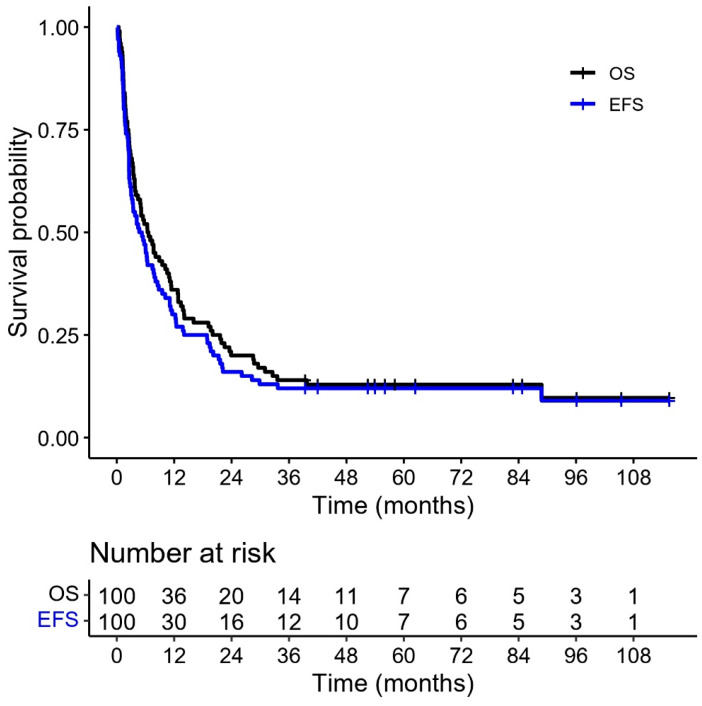
Kaplan–Meier curves for overall and event-free survival (OS and EFS) for whole cohort (N = 100). Ticks represent censoring of non-events.

**Figure 5:**
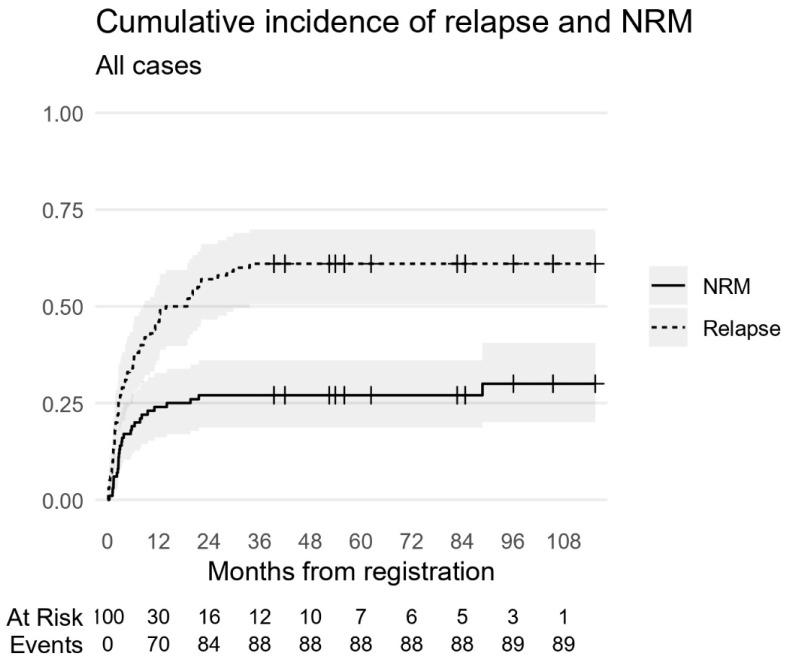
Cumulative incidence of relapse and related deaths and non-relapse mortality (NRM) for all cases (N = 100). Relapse and associated deaths occurred in 61 patients and represent primary event of interest, while NRM (toxic death) occurred in 28 and represents a competing event.

The 5-year EFS and OS were 12% and 13%, respectively (Figure 4). Sixty-one patients died of relapse or progressive disease. Twenty-eight patients died of non-relapse-related causes with a 5-year CIR of 61% (Figure 5). At the time of analysis, 11 patients were still alive. Eight patients were in CR, two had PR, and one patient had SD.

### 3.10. Causes of Death

A total of 89 patients died; of these, 61 deaths were due to progressive diseases or relapse, and 28 were from non-disease-related causes. Twenty-five patients died of septic shock. Thirteen of them had bloodstream infection: seven had Klebsiella pneumoniae (two patients had extremely drug-resistant infection, and one patient had multidrug-resistant infection), two patients had Escherichia coli, two patients had Stenotrophomonas maltophilia, and two patients had Candida albicans. See Table S3. Four patients died of necrotizing enterocolitis, two from pneumonia with respiratory failure, and one from CNS infection. Five patients died outside the hospital from an unknown infection.

Other non-disease-related mortality causes of death included secondary acute myeloid leukemia (AML), as seen in one patient; one case of extensive CNS thrombosis; and one case of chemotherapy-induced hyponatremia.

## 4. Discussion

In this study, we analyzed 100 ATRT patients using different prognostic factors, including age, primary site, metastatic status, extent of surgical resection, and molecular subgroups, and correlated these factors with survival. Sixty-nine patients were tested for molecular subtype using DNA methylation analysis.

Age had a significant impact on the outcome, as patients with an age <1 year had worse EFS and OS compared to older patients (1 to <3 years and >3 years), as none of the patients <1 year were alive at 5 years. These findings are consistent with data from the European Rhabdoid Registry (EU-RHAB), which reported a 5-year OS of 16.7 ± 5.7% for patients <1 year of age and 45.3 ± 6% for patients > 1 year of age [17]. In contrast, data reported by the children’s oncology group ACNS0333 trial showed that younger patients <36 months of age were not at a higher risk of death [18].

In this study, metastatic disease had a negative impact on the EFS, OS, and risk of relapse, matching with the results of Frühwald et al., who reported that metastatic disease was a negative prognostic factor with a 5-year OS of 16.9% for M+ patients vs. 43% for M0 patients (*p* = 0.0001) [17].

Upadhyaya et al. showed that infants and children with M0 disease had a 5-year progression-free survival (PFS) and OS of 31.4% and 43.9% for infants and 72.7% and 81.8% for children, respectively. In contrast, the 5-year OS for infants and children with M+ disease was 0% and 25%, respectively (*p* < 0.001) [3].

On the contrary, the ACNS0333 study reported that metastatic disease did not show a significant impact on OS (*p* = 0.58) [18]. These results may be due to the intensified approach using surgery, induction with high-dose methotrexate, tandem HSCT, and radiotherapy, which nullified the negative impact of different prognostic factors.

In our cohort treated according to the Dana-Farber Cancer Institute modified IRS-III protocol, four patients with metastatic disease who were younger than 3 years received focal radiation therapy. All of them experienced relapse; two had both distant and local relapse, one had distant relapse, and one had local relapse. However, based on small numbers, the current approach for infants with metastatic disease (focal radiation therapy) appears insufficient for this high-risk group, and additional treatment modalities are needed. Interesting data from the COG ACNS0333 trial, which replaced full-dose craniospinal radiation therapy with intensified induction followed by tandem transplant and lower-dose CSI (23.4 Gy), may improve the outcome for this subset of patients [18]. This is consistent with Upadhyaya et al., who reported that even children with non-metastatic ATRT may benefit from postoperative CSI and adjuvant chemotherapy [3].

Our data showed that patients with postoperative residual disease had inferior EFS and OS with an increased risk of relapse, which is consistent with the original report from Chi et al., who reported that the extent of resection had a significant impact on both PFS and OS (*p* = 0.008 and *p* = 0.004, respectively) [10]. However, in their 2024 updated abstract, the authors reported that the extent of resection no longer had an impact on outcomes [19]. This suggests that the effect of the degree of resection may diminish as data from longer follow-ups on survivors becomes available.

Desai et al. reported on a retrospective analysis of 60 patients with ATRT from different centers, including Dana Farber data. They found that neither patient age, extent of resection, nor metastatic status had a significant impact on survival [19].

Our experience with the molecular classification of the ATRT tissue samples highlights a critical issue associated with the evolving nature of classifier versions and their impact on diagnostic consistency. However, changes are primarily seen in samples reporting low scores, which by definition are not reliable. We observed a shift from ATRT to PB despite a confident score in the 12.5 version. Such discrepancies can present a challenge for longitudinal studies, where maintaining a consistent cohort definition is essential for unbiased clinical correlation. Hence, our findings support a diagnostic approach that does not rely solely on a single classifier score. Instead, integrating methylation profiling with supporting molecular and pathological data is essential, particularly for samples with low calibrated scores or ambiguous classification.

In our cohort, patients with a TYR signature were significantly younger than those with ATRT-MYC and ATRT-SHH, with a median age of 0.97 years, 2.44 years, and 1.91 years, respectively. ATRT-TYR is exclusively presented in the infra-tentorial region, whereas ATRT-SHH and MYC were exhibited in both supra-tentorial and infra-tentorial locations. All patients with spinal involvement were classified as ATRT-MYC.

These findings were consistent with Ho et al., where the ATRT-TYR subgroup predominantly occurred in younger patients, with a median age of diagnosis of 12 months. In contrast, ATRT-MYC tumors were more commonly found in older children, with a median age of 27 months. Most ATRT-TYR tumors were infra-tentorial, while ATRT-SHH and ATRT-MYC predominantly arose in the supra-tentorial region. Notably, all spinal ATRTs were within the ATRT-MYC subgroup [8].

We attempted to correlate molecular subgroups with outcome, as there is no clear consensus in the literature regarding the prognostic impact of epigenetic subgroups. The three molecular subgroups had no impact on survival or the cumulative incidence of relapse. For the MYC, SHH, and TYR subgroups, the 5-year EFS was 18%, 12%, and 7.7%, respectively; the 5-year OS was 18%, 15%, and 0%; and the 5-year cumulative incidence for relapse was 53%, 68%, and 46%, respectively.

Similarly to these findings, the ACNS 0333 trial did not report a significant impact of molecular subgroup on survival, where the 4-year OS for the SHH, TYR, and MYC subgroups was 56%, 41%, and 27%, respectively, and the trial attributed the lack of difference to the small sample sizes of each group [18].

In the EU-RHAB registry, high-risk patients (non-TYR subgroup and age under 1 year) had a 5-year OS of 0% compared to 71% for patients with standard risk (ATRT-TYR disease and age >1 years) and 32% for intermediate-risk patients (<1 y + TYR or ≥ one y + non-TYR) (*p* < 0.05) [17].

Based on these different data sets, including ours, there is still no clear consensus on the prognostic impact of epigenetic subgrouping, which needs more collaborative work with a larger number of patients. The tyrosine kinase inhibitors (TKIs) dasatinib and nilotinib displayed selective toxicity to ATRT-MYC cell lines, and dasatinib significantly improved survival in an intracranial orthotopic xenograft model. The EZH2 inhibitor tazemetostat has been tested in relapsed or refractory SMARCB1-deficient tumors, and a preliminary report suggested that a direct toxic effect on the SHH cell line may be helpful in this particular subtype [20].

The 5-year EFS and OS for our entire cohort were 12% and 13%, respectively. Desai et al. reported on the updated survival of the ATRT cohort using the Dana Farber protocol, where the 5-year PFS and OS were 30% and 38%, respectively [19].

Our cohort had a high rate of non-relapse mortality (n = 28) with a high rate of infection-related mortality (n = 25). At the same time, Chi et al. reported one death due to sepsis with pneumococcal infection [10]. This may indicate that lengthy and intensified treatment regimens need to be revisited despite the aggressive supportive measures applied in our center.

## 5. Conclusions

Molecular subgrouping by DNA methylation did not confer survival differences, underscoring the need for larger collaborative studies to clarify its prognostic role. The high rate of treatment-related mortality, predominantly infection-related, highlights the challenges of applying lengthy, dose-intensive regimens in resource-limited settings. These findings support the need for treatment adaptation in such contexts, favoring shorter, risk-adapted, and intensified strategies such as the COG 0333 approach with short intensified induction followed by consolidation with high-dose chemotherapy (HDCT), and risk-adapted radiation therapy may be more suitable in our setup, combined with robust supportive care to balance efficacy with safety.

## Figures and Tables

**Table 1:** The baseline clinical and molecular characteristics of patients for the whole cohort.

Variable	N (%)
Total	100 (100%)
Sex	
Female	41 (41%)
Male	59 (59%)
Age group	
<1 year	28 (28%)
1 to <3 years	46 (46%)
≥3 years	26 (26%)
Primary site	
Supra-tentorial	41 (41%)
Infra-tentorial	55 (55%)
Spine	4 (4%)
M stage	
M0	53 (53%)
M+	39 (39%)
Mx	8 (8%)
Extent of resection	
GTR/NTR	40 (40%)
STR/bx	60 (60%)
Molecular subgroup	
MYC	17 (17%)
SHH	34 (34%)
TYR	13 (13%)
Not available	36 (36%)

Abbreviations: GTR: gross total resection; NTR: near-total resection; STR: subtotal resection; bx: biopsy.

**Table 2:** Prognostic variables for overall survival (OS) using uni- and multivariable Cox regression.

	Univariable			Multivariable		
	HR	95% CI	*p*-Value	aHR	95% CI	*p*-Value
Age group						
<1 year	—	—		—	—	
1 to <3 years	0.6	0.4, 0.97	0.04	0.65	0.4, 1.1	0.1
≥3 years	0.51	0.3, 0.90	0.02	0.6	0.3, 1.1	0.1
Sex						
Female	—	—				
Male	1.1	0.7, 1.7	0.7			
Primary site						
Supra	—	—		—	—	
Infra/Spine	1.5	0.99, 2.35	0.05	1.2	0.7, 1.9	0.5
M stage						
M0	—	—		—	—	
M+	2.7	1.7, 4.3	<0.001	2.3	1.4, 3.95	0.002
Mx	2.35	1.1, 5.1	0.03	1.8	0.8, 4.15	0.14
Extent of resection						
GTR/NTR	—	—		—	—	
STR/bx	1.7	1.1, 2.6	0.02	1.2	0.7, 2.02	0.5
Molecular subgroup						
MYC	—	—				
SHH	0.9	0.4, 1.6	0.6			
TYR	1.4	0.65, 2.98	0.4			

Abbreviations: HR: hazard ratio; aHR: adjusted hazard ratio; CI: confidence interval; GTR: gross total resection; NTR: near-total resection; STR: subtotal resection; bx: biopsy.

**Table 3:** Prognostic variables for event-free survival (EFS) using uni- and multivariable Cox regression.

	Event-Free Survival (EFS)					
	Univariable			Multivariable		
	HR	95% CI	*p*	aHR	95% CI	*p*
Age group						
<1 year	—	—		—	—	
1 to <3 years	0.6	0.4, 0.9	0.024	0.6	0.4, 1.0	0.056
≥3 years	0.5	0.3, 0.8	0.011	0.5	0.3, 1.0	0.047
Sex						
Female	—	—				
Male	1.1	0.7, 1.6	0.8			
Primary site						
Supra	—	—		—	—	
Infra/Spine	1.6	1.0, 2.4	0.05	1.2	0.7, 1.8	0.6
M stage						
M0	—	—		—	—	
M+	2.8	1.8, 4.3	<0.001	2.3	1.4, 3.9	0.002
Mx	2.4	1.1, 5.1	0.027	2.0	0.9, 4.5	0.1
Extent of resection						
GTR/NTR	—	—		—	—	
STR/bx	1.7	1.1, 2.6	0.018	1.3	0.8, 2.2	0.3
Molecular subgroup						
MYC	—	—				
SHH	0.95	0.5, 1.8	0.9			
TYR	1.5	0.7, 3.2	0.3			

Abbreviations: HR: hazard ratio; aHR: adjusted hazard ratio; CI: confidence interval; GTR: gross total resection; NTR: near-total resection; STR: subtotal resection; bx: biopsy.

**Table 4:** Prognostic variables for the incidence of relapse, accounting for competing risks (non-relapse mortality) in a Fine–Gray sub-distribution hazards model.

	Univariable			Multivariable		
	HR	95% CI	*p*-Value	aHR	95% CI	*p*-Value
Age group						
<1 year	—	—				
1 to <3 years	0.8	0.4, 1.4	0.4			
≥3 years	0.9	0.5, 1.8	0.7			
Sex						
F	—	—				
M	1.2	0.7, 1.9	0.5			
Primary site						
Supra	—	—				
Infra/Spine	1.0	0.6, 1.6	>0.9			
M-stage						
M0	—	—		—	—	
M+	4.1	2.5, 7.0	<0.001	3.3	1.9, 5.7	<0.001
Mx	1.4	0.5, 4.2	0.6	1.5	0.5, 4.8	0.48
Extent of resection						
GTR/NTR	—	—		—	—	
STR/bx	2.8	1.6, 4.9	<0.001	1.8	0.9, 3.5	0.08
Molecular subgroup						
MYC	—	—				
SHH	1.3	0.6, 2.9	0.5			
TYR	0.8	0.3, 2.2	0.6			

Abbreviations: HR: hazard ratio; aHR: adjusted hazard ratio; CI: confidence interval; GTR: gross total resection; NTR: near-total resection; STR: subtotal resection; bx: biopsy; CI: cumulative incidence.

## Data Availability

The raw data supporting the conclusions of this article will be made available by the authors on request.

## Supplementary Materials

The following supporting information can be downloaded at https://www.mdpi.com/article/10.3390/cancers17183077/s1, Table S1. Common toxicities (CTCAE) by treatment phase; Table S2. Patient characteristics by molecular subgroup; Table S3. Types of bloodstream infection and resistance pattern; Figure S1. Roadmap for treatment protocol for patients with ATRT; Figure S2. Cumulative incidence of relapse and related deaths (a) by metastatic stage at baseline and (b) by extent of surgical resection.
