# Supplementary material for: Clinical and Molecular Characteristics of 100 Atypical Teratoid Rhabdoid Tumor Patients from Low- and Middle-Income Countries
Source: Cancers (Basel). 2025 Sep 20;17(18):3077. doi: 10.3390/cancers17183077 (PMC12468553; doi:10.3390/cancers17183077)
Supplement: Supplementary file 1 [file cancers-17-03077-s001.zip › cancers-3838139-supplementary.pdf]

cancers

MDPI

Supplementary Materials: Clinical and Molecular Characteristics of 100 Atypical Teratoid Rhabdoid Tumor Patients From Low- and Middle-Income Countries

Noha A. Ismail [†,1], Shaimaa Aboubakr [†,1], Amal Mossab [2], Eslam Maher [3,4], Hanafy Hafez [1,5], Hala Taha [6,7], Dina Yassin [8], Amal Refaat [9,10], Mohamed S.Zaghloul [11.12], Mohamed EI- Beltagy [13,14], Abdelrahman Enayat [13.14], Volker Hovestadt [15,16], Olfat Ahmed [17,18], Mark w. Kieran [1], Ahmed El hemaly [1,5 *], Shahenda EI- Naggar [2*] and Alaa El-Haddad [1,5]

Table S1. Common toxicities (CTCAE) by treatment phase.

**Toxicity**, N = 100	**Induction (Week 1-6)** (Number at risk = 78)	**Consolidation (Week 7-18)** (Number at risk = 47)	**Maintenance (Week 19-End)** (Number at risk = 38)
**Anemia**			
Grade 3	76 (76%)	41 (41%)	31 (31%)
Grade 4	1 (1.0%)	0 (0%)	0 (0%)
No	1 (1.0%)	6 (6.0%)	7 (7.0%)
Not evaluable	22 (22%)	53 (53%)	62 (62%)
**Febrile Neutropenia**			
Grade 3	57 (57%)	42 (42%)	31 (31%)
Grade 4	8 (8.0%)	1 (1.0%)	1 (1.0%)
Grade 5	13 (13%)	1 (1.0%)	2 (2.0%)
No	0 (0%)	3 (3.0%)	4 (4.0%)
Not evaluable	22 (22%)	53 (53%)	62 (62%)
**Hypokalemia**			
Grade 3	4 (4.0%)	11 (11%)	7 (7.0%)
Grade 4	61 (61%)	12 (12%)	8 (8.0%)
No	13 (13%)	24 (24%)	23 (23%)
Not evaluable	22 (22%)	53 (53%)	62 (62%)
**Hypomagnesemia**			
Grade 3	22 (22%)	6 (6.0%)	5 (5.0%)
Grade 4	14 (14%)	8 (8.0%)	1 (1.0%)
No	42 (42%)	33 (33%)	32 (32%)
Not evaluable	22 (22%)	53 (53%)	62 (62%)
**Hyponatremia**			
Grade 3	20 (20%)	5 (5.0%)	—
Grade 4	10 (10%)	1 (1.0%)	—
Grade 5	1 (1.0%)	0 (0%)	—
No	46 (46%)	41 (41%)	—
Not evaluable	23 (23%)	53 (53%)	—
**Hypocalcemia**			
Grade 3	28 (28%)	8 (8.0%)	0 (0%)
Grade 4	16 (16%)	4 (4.0%)	2 (2.0%)
No	34 (34%)	35 (35%)	36 (36%)
Not evaluable	22 (22%)	53 (53%)	62 (62%)
**Nephrotoxicity**			
Cystitis Grade 3	2 (2.0%)	—	—

**Toxicity,** N = 100	**Induction (Week 1-6)** (Number at risk = 78)	**Consolidation (Week 7-18)** (Number at risk = 47)	**Maintenance (Week 19-End)** (Number at risk = 38)
No	79 (79%)	—	—
Not evaluable	19 (19%)	—	—
**GIT toxcicity**			
Grade 3	11 (11%)	3 (3.0%)	2 (2.0%)
Grade 4	1 (1.0%)	0 (0%)	0 (0%)
Grade 5	1 (1.0%)	0 (0%)	0 (0%)
No	65 (65%)	44 (44%)	36 (36%)
Not evaluable	22 (22%)	53 (53%)	62 (62%)
**Type of GIT toxicity**			
diarrhea Grade 3	6 (6.0%)	0 (0%)	1 (1.0%)
oral mucositis Grade 3	8 (8.0%)	3 (3.0%)	1 (1.0%)
vomiting Grade 3	1 (1.0%)	0 (0%)	0 (0%)
diarrhea Grade 4	1 (1.0%)	0 (0%)	0 (0%)
diarrhea Grade 5	1 (1.0%)	0 (0%)	0 (0%)
**neurotoxicity**			
DCL Grade4	2 (2.0%)	—	0 (0%)
Seizures Grade3	0 (0%)	1 (1.0%)	0 (0%)
occlumotor nerve Grade3	4 (4.0%)	—	1 (1.0%)
periphral motor neuropathy Grade3	0 (0%)	—	1 (1.0%)

Abbreviations:DCL:disturbed conscious level, GIT:gastrointestinal tract.

Table ***S*2.** Patient characteristics by molecular subgroup.

	**Overall**, N = 64	**MYC**, N = 17	**SHH**, N = 34	**TYR**, N = 13	**P-value**
**Sex**					0.4
Female	25 (39%)	7 (41%)	15 (44%)	3 (23%)	
Male	39 (61%)	10 (59%)	19 (56%)	10 (77%)	
**Age**					0.010
Mean (SD)	2.33 (2.16)	2.72 (1.79)	2.59 (2.54)	1.14 (0.74)	
Median (IQR)	1.84 (0.97, 2.93)	2.44 (1.53, 3.00)	1.91 (1.05, 2.96)	0.97 (0.58, 1.36)	
Range	0.29, 11.62	0.70, 7.95	0.36, 11.62	0.29, 3.05	
**Site**					0.029
Supra	25 (39%)	9 (53%)	15 (44%)	1 (7.7%)	
Infra/Spine	39 (61%)	8 (47%)	19 (56%)	12 (92%)	
**M stage**					>0.9
M0	38 (59%)	10 (59%)	19 (56%)	9 (69%)	
M+	22 (34%)	6 (35%)	13 (38%)	3 (23%)	
Mx	4 (6.2%)	1 (5.9%)	2 (5.9%)	1 (7.7%)	
**Extent of resection**					0.14
GTR/NTR	31 (48%)	5 (29%)	20 (59%)	6 (46%)	
STR/bx	33 (52%)	12 (71%)	14 (41%)	7 (54%)	
**W6 response**					0.4
CR/CCR	25 (57%)	6 (50%)	15 (60%)	4 (57%)	
PR	10 (23%)	5 (42%)	3 (12%)	2 (29%)	

SD	5 (11%)	0 (0%)	4 (16%)	1 (14%)
PD	4 (9.1%)	1 (8.3%)	3 (12%)	0 (0%)

Abbreviations: SD: standard deviation, IQR:interquartile range GTR: gross total resection, NTR: near total resection, STR: subtotal resection, bx: biopsy, CR:complete remission, PR:partial response, SD: stable disease, PD:progressive disease

Table S3. Types of blood stream infection and resistance pattern.

Type of Blood stream infection (N=13)	Type of resistance pattern
Klebsiella pneumonia(N=7)	Extremely drug-resistant (N=2)<br>Multidrug-resistant (N=1)<br>ESBL (N=4)
Escherichia coli (N=2)	ESBL
Stenotrophomonas maltophilia (N=2)	Multidrug-resistant
Candida Albicans(N=2)	NA

Abbreviations: NA: not applicable, ESBL: Extended-Spectrum Beta-Lactamase.

Pre-Irradiation/Chemo-Radiation/Post Radiation induction Chemotherapy

	Pre-Irradiation						EVALUATION	2nd SURGERY	Chemo-Radiation						Post-Radiation		EVALUATION
Week / Day	1	2	3	4	5	6			7	8	9	10	11	12	13	16	
1	IT, V, P	IT, V	V	IT, V, P, E	V	V			IT, V, P, E	V	V	V, P, E	V	V	IT, V, D, C	V, A, C	
2	D, C			E, D					E, C*			E, C*			C, D	A, C	
3	D, C			E, D					E			E			C	A, C	
4	C															A	
5																A	
									** RADIATION								

If initial CSF cytology is +ve, give IT weekly until two consecutive CSF cytology are –ve, then continue IT as per roadmap.

Patients < 1 yr start CTH (**except IT**) at **50%** dosing, If tolerated, escalate to **75%** and then to **100%** with subsequent cycles.

Maintenance Chemotherapy

Week / Day	19	23	27	30	EVALUATION	33	36	39	42	EVALUATION
1	IT, A*, T	A*, T	IT, V, D, C	V, A, C		IT, V, D, C	V, A, C	IT, V, D, C	V, A, C	
2	T	T	D, C	A, C		D, C	A, C	D, C	A, C	
3	T	T	C	A, C		C	A, C	C	A, C	
4	T	T		A			A		A	
5	T	T		V, A			V, A		V, A	

No CSI & No Mediastinal Radiation

Doxorubicin Cont. Therapy

Week / Day	45	48	51	EVALUATION
1	IT, V, C, D	C, A, V	IT, V, C, D	
2	C, D	C, A	C, D	
3	C	C, A	C	
4		A		
5		A, V		

CSI or Mediastinal Radiation

Non Doxorubicin Cont. Therapy

Week / Day	45	48	51	EVALUATION
1	IT, V, C, A	C, A, V	IT, V, A, C	
2	A, C	C, A	A, C	
3	A, C	C, A	A, C	
4	A	A	A	
5	A,V	A, V	A, V	

****For M0 all ages OR M+ < 3 years:** Conformal RTH | ****For M+ AND ≥ 3 years:** CSI | **Evaluation:** MRI Brain & Spine

Vincristine (V) 2 mg/m^2 (max 2 mg)
Cisplatin (P) 90 mg/m^2
Doxorubicin (D) 30 mg/m^2/day
Temozolomide (T) 200 mg/m^2 if conformal RTH **OR** 150 mg/m^2 if CSI

Cyclophosphamide (C) 300mg/m^2/day
Cyclophosphamide (C*) 600 mg/m^2
Actinomycin-D (A) 0.015 mg/kg/day
Actinomycin-D (A*) 1.2 mg/m^2 (max 2.5 mg)
Etoposide (E) 100 mg/m^2

Intrathecal (IT)
Methotrexate 15 mg/m^2 (max 15 mg)
Hydrocortisone 30 mg/m^2 (max 30 mg)
Cytarabine 60 mg/m^2 (max 60 mg)

Figure S1. Roadmap for treatment protocol for patients with ATRT, CSI: cranio-spinal irradiation, CSF: cerebrospinal fluid, Cont. Therapy: continuation therapy

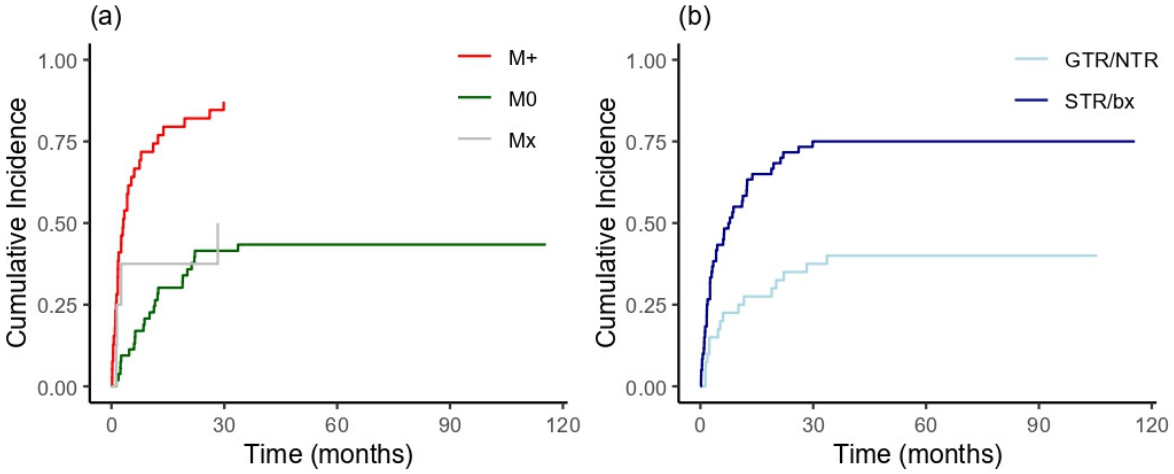

Figure S2. Incidence of relapse & related deaths via the cumulative incidence function, (a) by metastatic stage at baseline, and (b) by extent of surgical resection.
